# Gut microbiota alterations and associations with nutrients in children with celiac disease

**DOI:** 10.1002/fsn3.4337

**Published:** 2024-09-12

**Authors:** Yasemin Ertaş Öztürk, Efsun Karabudak, Ödül Eğritaş Gürkan, Buket Dalgıç

**Affiliations:** ^1^ Department of Nutrition and Dietetics, Faculty of Health Sciences Ondokuz Mayıs University Samsun Türkiye; ^2^ Department of Nutrition and Dietetics, Faculty of Health Sciences Sanko University Gaziantep Türkiye; ^3^ Department of Pediatric Gastroenterology and Hepatology, Faculty of Medicine Gazi University Ankara Türkiye

**Keywords:** celiac disease, dietary fat, gluten‐free diet, microbiota

## Abstract

Celiac disease is a chronic inflammatory condition that is not well understood in relation to the microbiome. Our objective was to demonstrate changes in the microbiota and the relationships between nutrients in children with celiac disease (CD) who followed a gluten‐free diet (GFD). A group of 11 children who were recently diagnosed with CD, ranging in age from 3 to 12, were monitored for a period of 6 months. GFD is designed based on the individual's specific energy and nutrient needs, with strict control over dietary adherence. Food consumption, blood, and fecal samples were taken. Fecal samples were put through 16s rRNA sequencing. Microbial modifications were demonstrated using alpha diversity, beta diversity, nonmetric multidimensional scaling analysis (NDMS), *t*‐test, and metastats. Mean age was 6.4 ± 2.66 years and 54.5% were male participants. Serological parameters were negative after 6 months. Both unweighted (*p* = .019) and weighted (*p* = .021) Unifrac distances were higher before GFD, and differences were reliable according to NDMS analysis (stress = 0.189). The abundance of *Bacteroides ovatus* was increased (*p* = .014), whereas unidentified *Lachnospiraceae*, *Paeniclostridium*, *Paraclostridium Peptostreptococcus*, and *Dielma* were decreased after GFD (*p* < .001). Associations between nutrients and several genera and species were identified. The presence of genus *Bifidobacterium* and *Bifidobacterium adolescentis* was inversely associated with fat intake after GFD (*p* < .01). Microbiota changes became evident over a period of 6 months. The presence or absence of small bacteria may play a role in the development of CD. Modifying the children's dietary intake can potentially influence the microbial composition.

## Graphical abstract

Microbiota changes became evident over a period of 6 months. The presence or absence of small bacteria may play a role in the development of CD and the abundance of *Bacteroides ovatus* was increased after the treatment. Modifying the children's dietary intake can potentially influence the microbial composition.

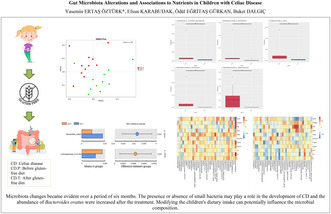

## 1 INTRODUCTION

Celiac disease (CD) is a chronic autoimmune condition of small intestine that is triggered by gluten‐containing food consumption, for example, wheat, rye, and barley, in people with genetic predisposition (Verdu & Schuppan, 2021). Epidemiological data suggest that CD is one of the prevalent lifelong diseases affecting almost 1% of worldwide with geographical differences (Lionetti et al., 2015). In Türkiye, the seroprevalence and biopsy‐proven prevalence of CD were found to be 2.42% and 0.47%, respectively, in children (Dalgic et al., 2011).

Because not all people with genetic predisposition suffer from CD (Verdu et al., 2015), the current evidence emphasizes that environmental factors are crucial in the development of the CD (Lebwohl et al., 2015). The environmental factors affecting the development of CD are unclear; however, cross‐talk between gluten and intestinal microbiota may have an important role in disease onset and progression (Chander et al., 2018).

The microbial pattern associated with CD exhibits significant variability based on current evidence. Alterations in different taxon levels in the gut microbiota of treated and untreated children with CD, increases in potential pathogenic microorganisms, and decreases in potentially beneficial species in the active period of the disease were shown (Akobeng et al., 2020; Pecora et al., 2020). Decreased abundances of the beneficial species such as *Bifidobacterium* and *Lactobacillus* or increase in the *Bacteroides*, *Escherichia*, or *Staphylococcus* species has been reported (Rossi et al., 2023). Schippa et al. suggested that present/absent situation of certain microorganisms such as *Escherichia coli*, *Bacteroides* spp. in duodenum could be important to understand the microbial alterations in CD rather than a microbiota pattern after the follow‐up (Schippa et al., 2010). The alterations can be changed due to applied analysis, sample site, and methodology (Lupu et al., 2023).

Gluten‐free diet (GFD) is the only effective treatment in CD that improves gastrointestinal symptoms (See et al., 2015). Children who adhere strictly to a gluten‐free diet typically have a rapid improvement in gastrointestinal symptoms associated with celiac disease, such as bloating, diarrhea, stomach discomfort, and weight loss. They also see an improvement in other symptoms beyond the digestive system, such as anemia, delayed puberty, and stomatitis. Poor dietary adherence is linked to inconsistent or nonexistent follow‐up (Mearin et al., 2022). Dietary compliance depends on the success of the education given to parents of children with CD. In Türkiye, GFD compliance was 73.3% in children (Esenyel et al., 2014). Full compliance with the diet in children remains at a low level of 58.0%. It is due to reasons such as the fact that gluten‐free preferences in the diet are more unpalatable, the diet limits eating outside, difficulties in accessing gluten‐free products, asymptomatic progression of the disease, social pressures, inadequacies in food labeling, cost, and transition to adolescence (MacCulloch & Rashid, 2014; Roma et al., 2010).

Removing gluten sources from the diet leads to changes in the food structure. In order to preserve the structure of special gluten‐free products, there is often a higher usage of carbohydrates and fats (Bascunan et al., 2017; Melini & Melini, 2019). However, alterations of the nutritional composition of GFD can be a confounding factor on the alterations of gut microbiota (Lee & Kao, 2017). GFD can influence gut microbiota composition, especially alter microbial activity that play roles in starch metabolism in healthy people (Bonder et al., 2016). However, there is limited study showing gut microbiota alterations along with associations between diet and microbiota in children with CD (Soheilian‐Khorzoghi et al., 2022; Zafeiropoulou et al., 2020). Therefore, we aimed to determine alterations of gut microbiota and the associations between microbiota and nutrients under a dietician controlled GFD treatment in children with CD in this study.

## 2 METHOD

### 2.1 Study protocol and subjects

**FIGURE 1:**
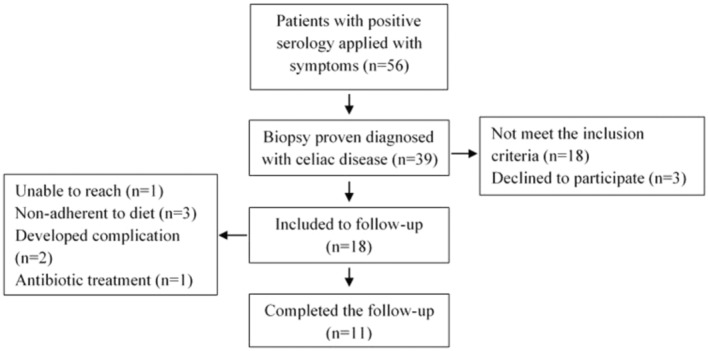
Flow chart of inclusion to follow‐up.

The inclusion process is shown in Figure 1. According to paired means power analysis (*Z*‐test), the sample size was adequate for conducting the present study (power: 0.99999, β: 0.00001, α: 0.05). Eleven newly diagnosed children with CD (male: 54.5%, female: 45.5%; mean age: 6.4 ± 2.66, ranged 3–12 years) were enrolled in this study at (Gazi University), Faculty of Medicine, Department of Pediatric Gastroenterology and Hepatology. CD were diagnosed with positive serology (positive tissue transglutaminase or antiendomysial antibody levels with normal total IgA levels) and duodenal biopsy through Marsh classification according to ESPGHAN criteria (Husby et al., 2012). Children with chronic illness and medicine, antibiotic, or probiotic users at least a month before the study was not included. Fecal samples were collected twice from CD patients before the implementation of GFD (active state/celiac disease patient group; abbreviated as CD.P in tables and figures) and after 6‐month follow‐up under GFD treatment (inactive state/celiac disease treatment group; abbreviated as CD.T in tables and figures). All collected fecal samples were stored at −80°C until DNA extraction. The study protocol was approved by the Clinical Research Ethics Committee of (Gazi University (date:22/01/2018 and number:24074710‐8/42)) and patients were enrolled in the study after obtaining informed consent from their parents.

### 2.2 Gluten‐free diet treatment

A trained dietician assessed the food consumption of each child by 3‐day food consumption records. As GFDs can lack certain macro‐ and micronutrients such as dietary fiber, plant protein, calcium, iron, magnesium, and thiamine (Vici et al., 2016), an individual diet was planned according to the energy and nutrient requirements of each child with compliance with Turkish Dietary Guideline recommendations (Başoğlu & Tek, 2016) and weekly controls were done to assess adherence. The controls were conducted by telephone communication with the caregiver in order to improve adherence to the GFD (gluten‐free diet). All dietary data were analyzed using the Nutrition Data System (BeBiS 7.0) software containing Turkish food composition tables. This system presents comprehensive data on both macro‐ and micronutrients once the food records entered, specifying the kind and quantity of each food item. Dietary information of children is shown in Table 2 before and under GFD treatment.

### 2.3 DNA extraction, sequencing, and library preparation

Bacterial DNA was extracted with QuickGene DNA tissue kit (Kurabo, Osaka/Japan) with the QuickGene‐Mini80 instrument (Kurabo, Osaka/Japan) according to the manufacturer's instructions. Briefly, 25‐mg fecal samples were added to homogenization tube with 250‐μL tissue lysis buffer and homogenized. Then, 25‐μL proteinase K were added and mixed for 60 min at 55°C. Then, samples were centrifuged at 15,000 × g for 10 min. The supernatants were transferred to a microtube and 180‐μL lysis buffer was added and mixed with vortex for 15 s and incubated at 70°C for 10 min. After incubation, 240‐μL ethanol (>99%) was added immediately and mixed with vortex for 15 s. Lysates were transferred to QuickGene cartridges and DNA binding, washing, and elution were accomplished through pressurization. Finally, DNA was obtained with elution volume of 200 μL. Quality of the DNA was assessed with Colibri Microvolume Spectrometer (Berthold Titertek Instruments, Germany) and Qubit® 2.0 Fluorometer (Thermo Fisher). Afterwards, sequencing and bioinformatics analyses were performed by Novogene (Wan Chai, Hong Kong). 16S rRNA genes of 16SV4/16SV3/16SV3‐V4/16SV4‐V5 regions were amplified by using specific forward (e.g., V4:515F‐GTGCCAGCMGCCGCGGTAA) and reverse (e.g., V4:806R‐GGACTACHVGGGTWTCTAAT) primers with the barcode. Polymerase chain reactions (PCR) were done with Phusion® High‐Fidelity PCR Master Mix (New England Biolabs). All PCR products were mixed with 1× loading buffer (contained SYB green) and electrophoresis was carried out on 2% agarose gel for detection. Later, PCR products were mixed in isodensity ratios and purified with Qiagen Gel Extraction Kit (Qiagen, Germany). The analysis was conducted in a single batch and executed to prevent any technical bias.

NEBNext Ultra DNA Library Pre® Kit for Illumina was used to prepare sequencing libraries by following the manufacturer's instructions. The library quality was assessed on the Qubit® 2.0 Fluorometer and Agilent Bioanalyzer 2100 system. Finally, the library was sequenced on an Illumina platform and 250 bp paired‐end reads were obtained.

### 2.4 Bioinformatics and statistical analysis

Using a unique barcode system for the samples, paired‐end reads were merged by FLASH (V1.2.7) (Magoč & Salzberg, 2011), and quality filtering was performed to obtain the high‐quality clean tags according to the QIIME (V1.7.0) (Caporaso et al., 2010). Sequences with ≥97% similarity were assigned to the same OTUs. In order to study phylogenetic relationship of different OTUs, and the difference of the dominant species in different groups, multiple sequence alignments were conducted using the MUSCLE software (Version 3.8.31) (Edgar, 2004). Alpha diversity indices (ACE, Chao1, Shannon, and Simpson) were calculated by QIIME from rarefied samples using richness and diversity indices of the bacterial community. Beta diversity on both weighted and unweighted Unifrac was calculated by QIIME software (Version 1.7.0). Nonmetric multidimensional scaling analysis (NDMS) was performed for a better representation of nonlinear biological data structure aiming at overcoming the flaws in methods based on linear model, including PCA and PCoA. A t‐test was performed to determine species with significant variation between groups at various taxon ranks. Species with significant intragroup variation were detected via metastats according to their abundance. The significance of observed abundance's differences among groups was evaluated via multiple hypothesis test for sparsely‐sampled features and false discovery rate. Clinical, biochemical, and dietary data of the children were represented as mean ± standard deviation. Paired sample *t*‐test was performed to show dietary differences. Pearson correlation analysis was performed to show the associations between microbiota and nutrients. The statistical significance level was set as *p* < .05. The SPSS statistical software package (Version 22.0) was used for the analyses.

## 3 RESULTS

Clinical and biochemical parameters of the children before and under GFD are shown in Table 1. CD serological biomarkers (anti‐tTG IgA and anti‐EMA IgA) of the children were negative after 6 months.

**TABLE 1:** Clinical and biochemical parameters of the children before and under gluten‐free diet.

Parameters	CD.P (*n* = 11)	CD.T (*n* = 11)
Age (years) (mean ± SD and range)	6.4 ± 2.66 (3–12)	–
Gender (male/female)	6/5	–
Type of delivery		
Vaginal	4 (36.4%)	–
Cesarean	7 (63.6%)	–
Type of feeding		
Breastfeeding	6 (54.5%)	–
Breastfeeding + formula	5 (45.5%)	–
Biochemical parameters (mean ± SD)		
Total IgA (mg/dL)	132.2 ± 13.57	N/A
Anti‐tTG IgA (U/mL) (*n* = 7)	84.5 ± 32.05	<10 (Negative)
Anti‐EMA IgA (U/mL) (*n* = 4)	124.8 ± 32.66	<10 (Negative)
Biopsy classification		
Marsh 3a	2 (18.2%)	N/A
Marsh 3b	5 (45.4%)	N/A
Marsh 3c	4 (36.4%)	N/A

Abbreviations: CD.P, Celiac disease patients before gluten‐free diet; CD.T, Celiac disease patients under gluten‐free diet; EMA, Endomysial antibody; IgA, Immunoglobulin A; N/A, Not available; tTG, Tissue transglutaminase.

In Table 2, meeting adequate intake for dietary energy, fiber, thiamine, and calcium of the children were similar before and under GFD (*p* > .05). Likewise, energy from carbohydrate, protein, and fat was not different (*p* > .05). Only energy percentage from sugar decreased under GFD (*p* = .013).

**TABLE 2:** Dietary energy and nutrient intake of children before and under gluten‐free diet (mean ± SD).

Dietary parameters	CD.P (*n* = 11)	CD.T (*n* = 11)	*p* Value
Energy (DRV %)	94.0 ± 19.19	99.2 ± 12.67	.348
Protein (Energy %)	13.2 ± 3.12	13.8 ± 2.99	.404
Fat (Energy %)	37.8 ± 3.63	36.3 ± 5.62	.400
Carbohydrate (Energy %)	48.8 ± 5.62	50.3 ± 8.10	.430
Sucrose (Energy %)	9.9 ± 3.91	6.2 ± 2.12	.006*
Dietary fiber (DRV %)	105.5 ± 44.95	99.9 ± 31.65	.751
Thiamine (DRV %)	140.0 ± 59.32	126.8 ± 32.32	.546
Calcium (DRV %)	76.6 ± 43.63	82.7 ± 27.79	.644

Abbreviations: CD.P, Celiac disease patient group (before gluten‐free diet); CD.T, Celiac disease treatment group (under gluten‐free diet); DRV, Dietary reference value.

* *p* < .05.

**FIGURE 2:**
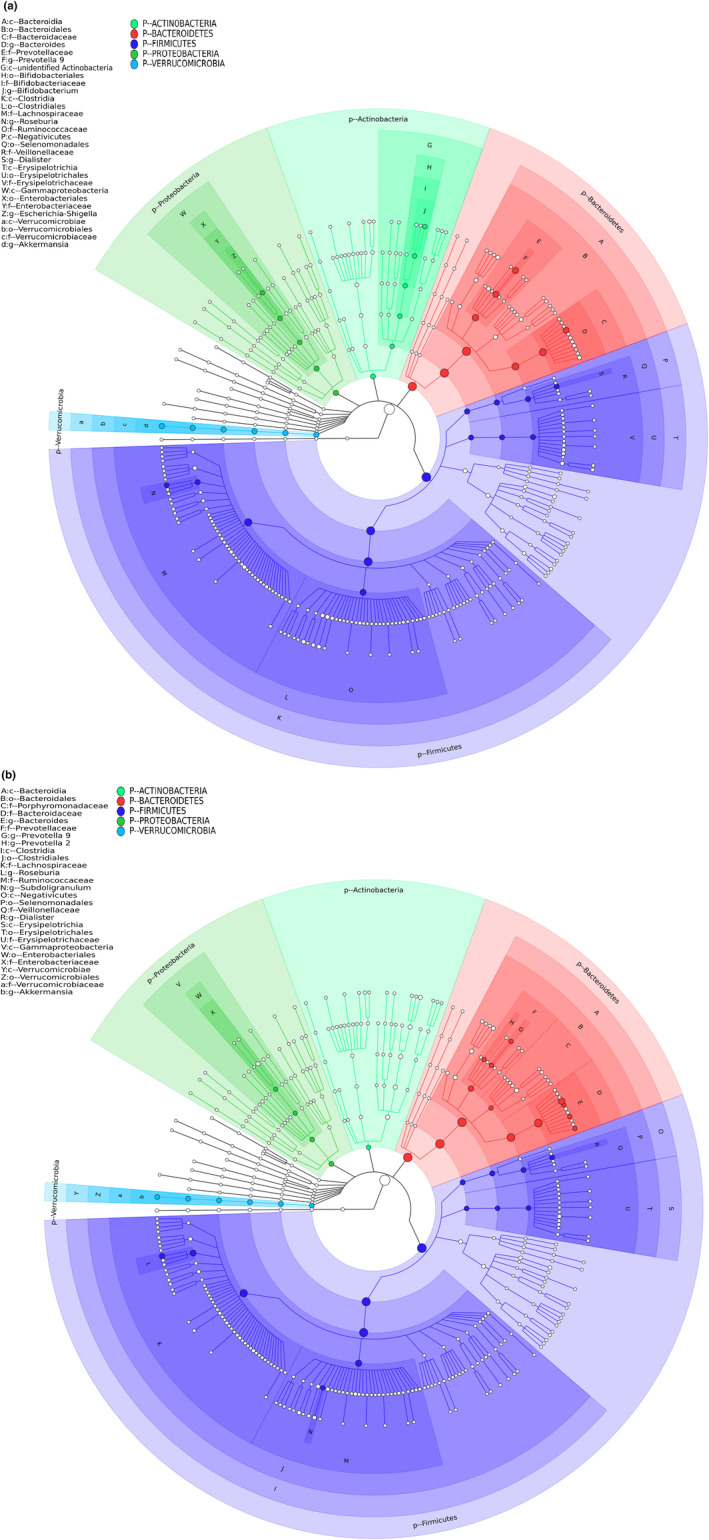
(a, b) Relative bacterial abundances of CD.P (a) and CD.T (b) groups. The size of the rings indicates the abundance of the dominant species. Each different color represents a different phylum. The taxon with the highest relative abundance of these phyla is plotted with small filled circles. CD.P, Celiac disease patient group (before gluten‐free diet); CD.T, Celiac disease treatment group (under gluten‐free diet).

In Figure 2a,b, relative abundance of taxon level was shown. *Firmicutes*, *Bacteroidetes*, *Actinobacteria*, *Proteobacteria*, and *Verrucomicrobia* were the dominant phylum. There was no significant difference between CD.P and CD.T at phylum level (*p* < .05).

**FIGURE 3:**
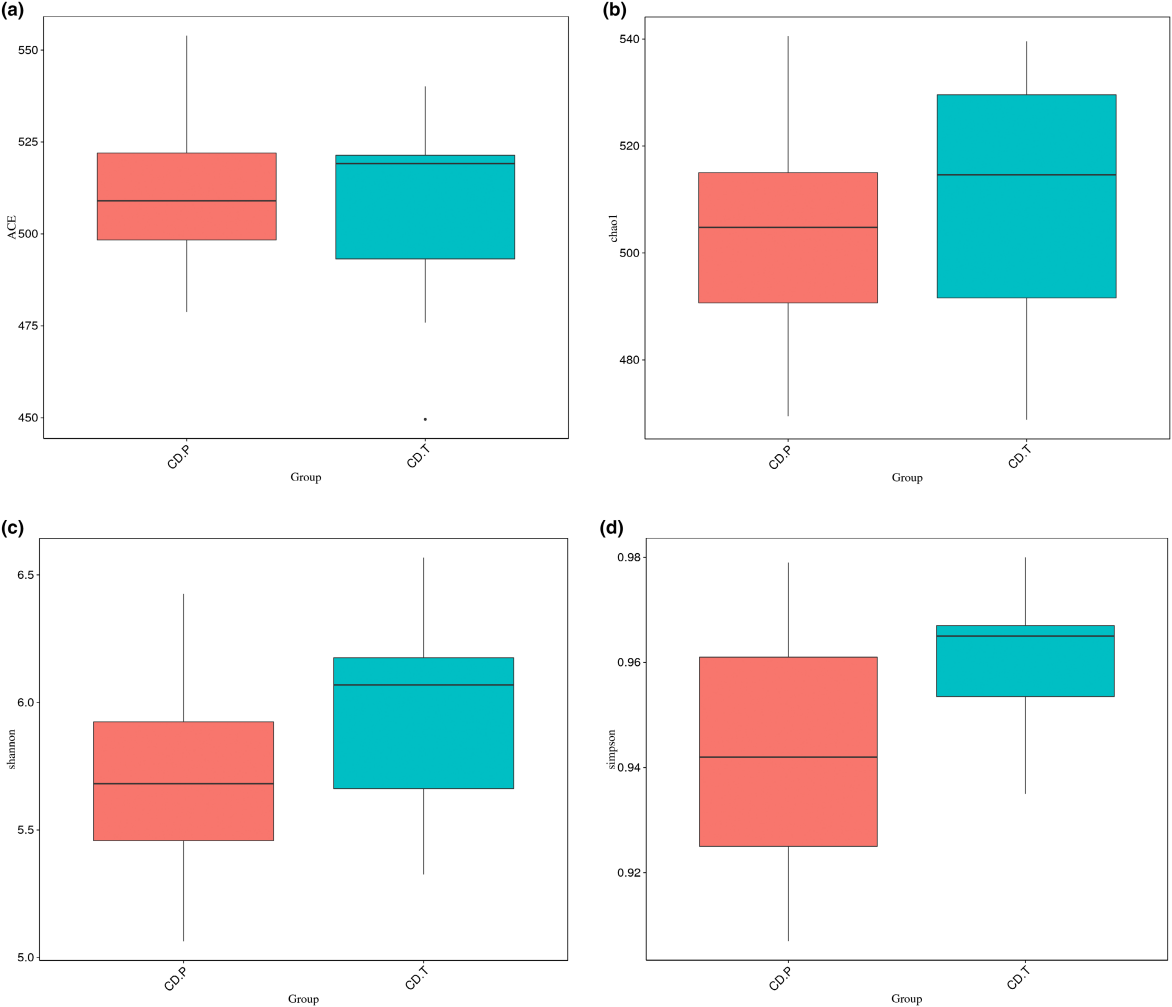
(a–d) Alpha diversity: ACE (a), Chao1 (b), Shannon (c), and Simpson (d) of CD.P and CD.T groups. CD.P, Celiac disease patient group (before gluten‐free diet); CD.T, Celiac disease treatment group (under gluten‐free diet).

**FIGURE 4:**
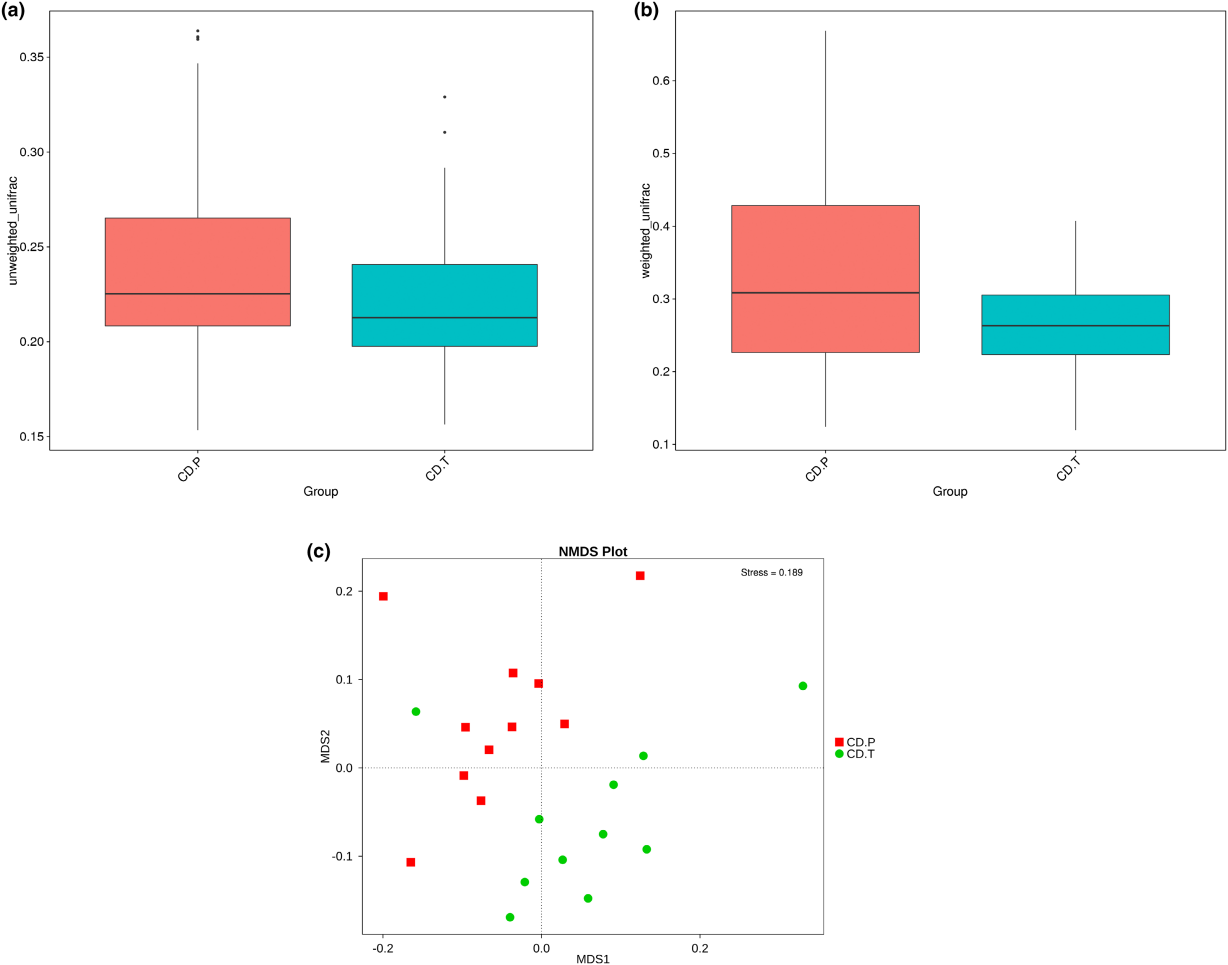
(a–c) Beta diversity: Unweighted unifrac (a) and unweighted unifrac (b) distances and nonmetric multidimensional scaling plot (c) of CD.P and CD.T groups. CD.P, Celiac disease patient group (before gluten‐free diet); CD.T, Celiac disease treatment group (under gluten‐free diet) of CD.P and CD.T groups.

No significant change between baseline and follow‐up was observed in alpha diversity indices as shown in Figure 3a–d. For beta diversity, unweighted and weighted Unifrac distance scores are shown in Figure 4a,b, respectively. In the active period of the disease, diversity was higher than the inactive period for both unweighted (*p* = .019) and weighted (*p* = .021). NDMS plot showed that separations between active and inactive states of disease started and stress factor less than 0.2 considered that NMDS is reliable (Figure 4c).

**FIGURE 5:**
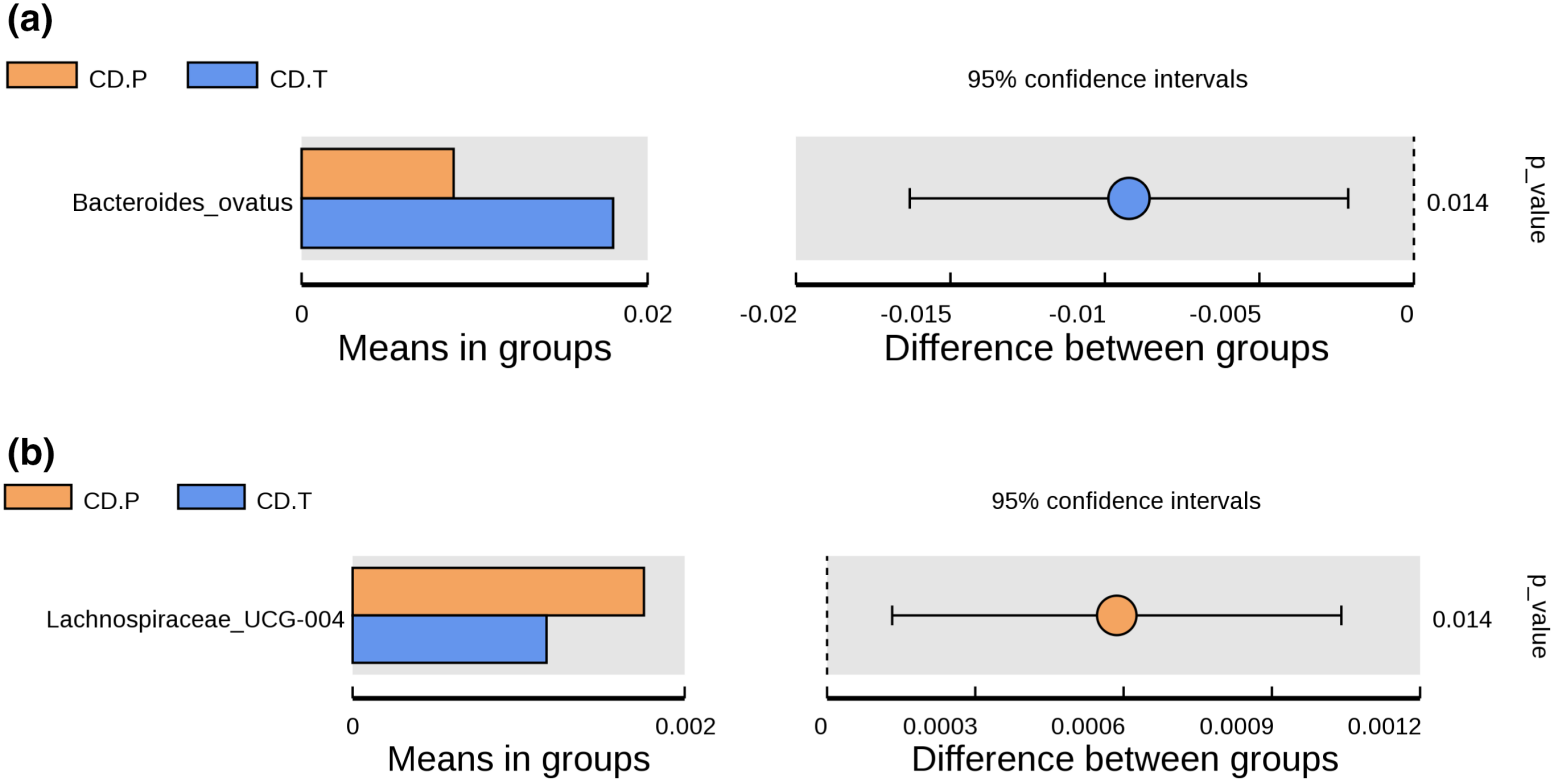
(a, b) Bacterial relative abundance differences between CD.P and CD.T groups. CD.P, Celiac disease patient group (before gluten‐free diet); CD.T, Celiac disease treatment group (under gluten‐free diet).

**FIGURE 6:**
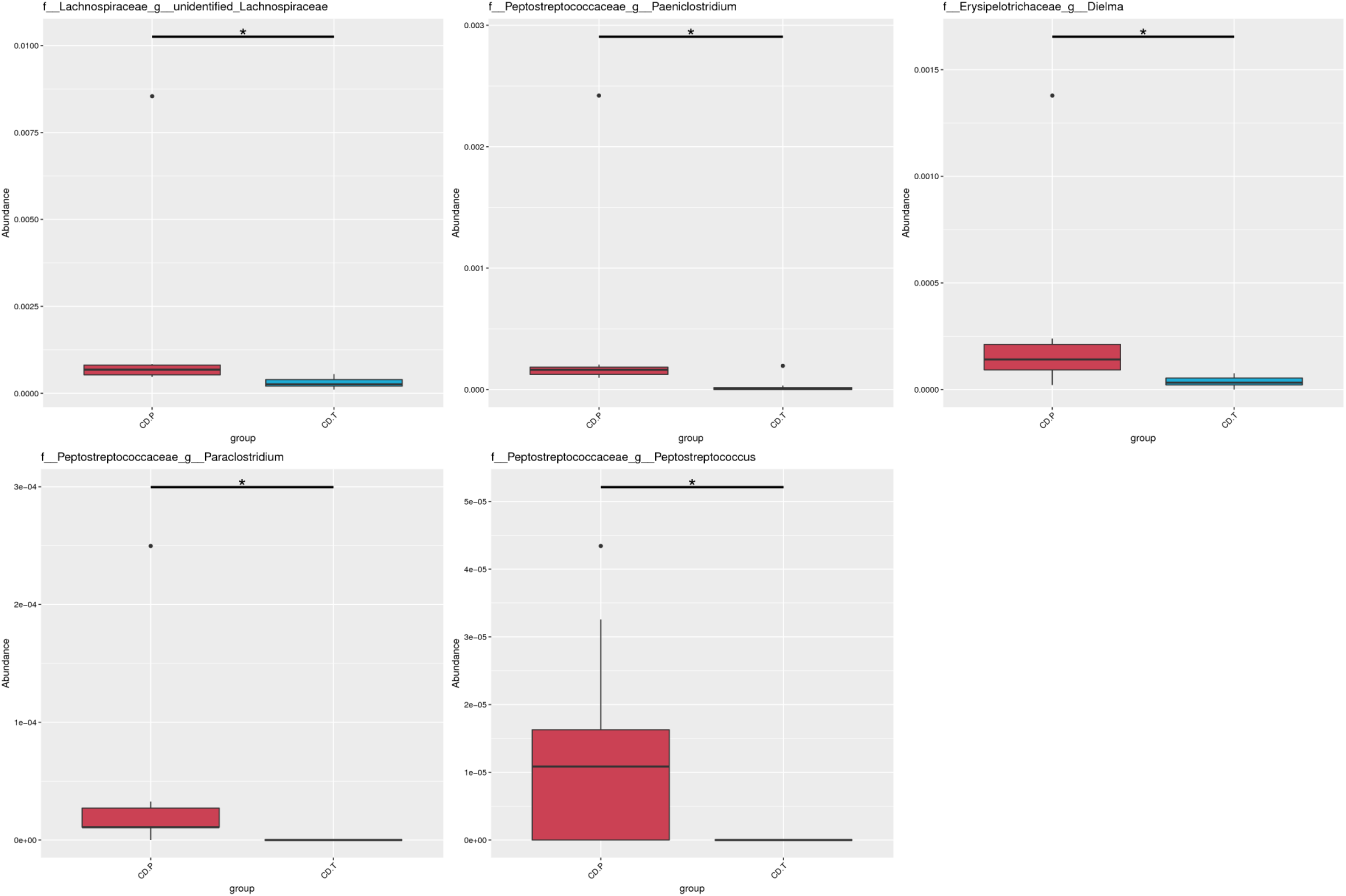
Relative abundances of minor bacteria: Unidentified *Lachnospiraceae*, *Paeniclostridium*, *Paraclostridium*, *Peptostreptococcus*, and *Dielma*. CD.P, Celiac disease patient group (before gluten‐free diet); CD.T, Celiac disease treatment group (under gluten‐free diet).

In Figure 5a, relative abundance of *Bacteroides ovatus* was significantly increased under GFD treatment (*p* = .014), whereas *Lachnospiraceae UCG‐004* were higher in the active state of disease (*p* = .014) (Figure 5b). Furthermore, *unidentified Lachnospiraceae*, *Paeniclostridium*, *Paraclostridium Peptostreptococcus*, and *Dielma* were lower in the inactive state of the disease response to GFD (*p* < .001) (Figure 6).

**FIGURE 7:**
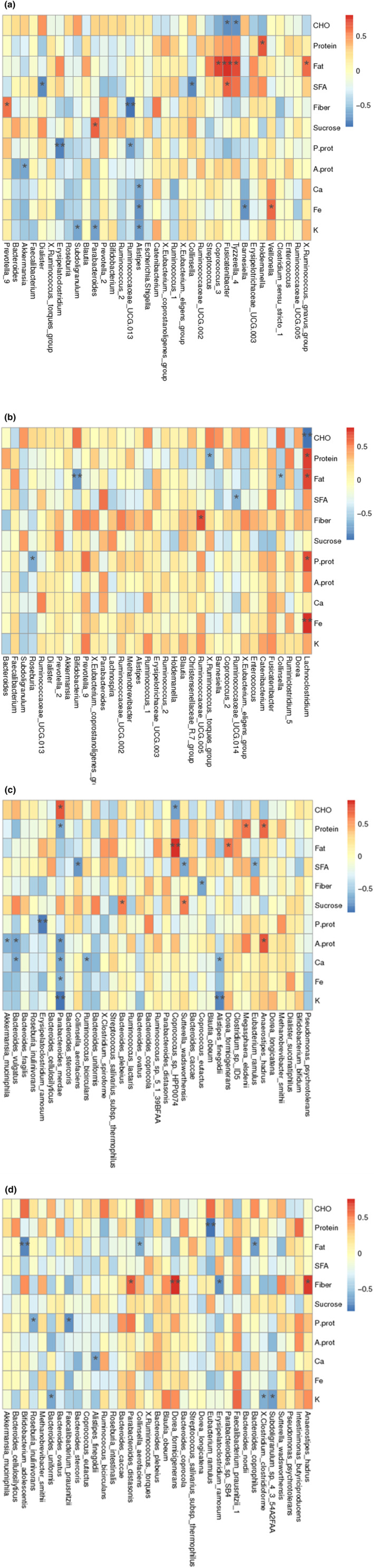
(a–d) Heat map showing associations between relative bacterial abundance at genus and species level with nutrients. Associations between genus and nutrients in CD.P (a), Associations between genus and nutrients in CD.T (b), Associations between species and nutrients in CD.P (c), Associations between species and nutrients in CD.T (d). Values are correlation coefficients. The heat map is color coded by correlation (red for positive and blue for negative correlations). (*) *p* < .05 and (**) *p* < .01. A.prot, Animal protein (g); Ca, Calcium (mg); CHO, Carbohydrate (energy%); Fat, Fat (energy%); Fe, Iron (mg); K, Potassium (mg); P.prot, Plant protein (g); Protein, Protein (energy%); SFA, Saturated fatty acids (energy%), Fiber (g); Sucrose, Sucrose (energy%).

In Figure 7a–d, the correlation heat map of the relationships between nutrients and relative abundance of bacterial genera and species of CD.P and CD.T is given in detail. The relationships differ according to groups.

## 4 DISCUSSION

In the present study, we followed 11 newly diagnosed children with CD for 6 months under a strictly controlled GFD treatment. The most important results of the study were: (1) Beta diversity analysis pointed out a difference in fecal microbiota composition between active (CD.P) and inactive (CD.T) states of the disease with a higher diversity in active state. NMDS plot suggested that the differences began to appear and reliable. (2) At genus level, unidentified *Lachnospiraceae*, *Paeniclostridium*, *Paraclostridium Peptostreptococcus*, and *Dielma* exist at the active state; but are almost absent at the inactive state of the disease. (3) We found that *Bacteroides ovatus* relative abundance increased under GFD treatment whereas *Lachnospiraceae UCG‐004* relative abundance decreased. (4) Microbiota and nutrient associations were diverse at the different states of the disease.

We found higher microbial diversity at the initial state of CD. Likewise, previous studies showed an increased diversity at the beginning of CD (Sanchez et al., 2013; Schippa et al., 2010). Schippa et al. suggested that mucosal alterations could be one of the reasons for the decreased diversity under GFD (Schippa et al., 2010). On the other hand, potential harmful bacterial growth may be another explanation for this pattern (Caminero et al., 2015; Sanchez et al., 2013). In the present study, we showed less abundant genera such as unidentified *Lachnospiraceae*, *Paeniclostridium*, *Dielma*, *Paraclostridium*, and *Peptostreptococcus* relative abundances were significantly higher in active state of disease. Unidentified *Lachnospiraceae* genus belongs to Firmicutes phylum, Clostridia class, Clostridiales order, and Lachnospiraceae family. In an animal study, unidentified *Lachnospiraceae* contributed to oxidative stress (Ma et al., 2019). Recently, the relative abundance of unidentified *Lachnospiraceae* has been reported in children with autism spectrum disorder (Jin et al., 2019) and type 2 diabetes mellitus (Kim et al., 2022). *Dielma* belongs to Firmicutes phylum, Erysipelotrichia class, Erysipelotrichales order, and Erysipelotrichaceae family. This family is closely related to inflammation, particularly increased TNF‐α levels, Crohn's disease, and colorectal cancer (Kaakoush, 2015). *Paeniclostridium*, *Paraclostridium*, and *Peptostreptococcus* genera are members of the Firmicutes phylum, Clostridia class, Clostridiales order, and Peptostreptococcaceae family. It has been reported that some strains of *Paeniclostridium sordellii* (*Clostridium sordellii*) can be found in the gastrointestinal tract with *Clostridium difficile* without causing any symptoms; however, it is mostly responsible for soft tissue infections (Scaria et al., 2015). Some strains belonging to the genus *Paraclostridium* have been shown to worsen pathogenesis by increasing TNF‐α, IL‐16, IL‐1, and IL‐17 expressions in the mouse‐induced ulcerative colitis model (Kutsuna et al., 2018). Likewise, the relative abundance of *Peptostreptococcus* has been reported to be high in children with Crohn's disease in a recent study (El Mouzan et al., 2018). In a previous study, species belonging to *Peptostreptococcus* were associated with dysbiosis in ulcerative colitis (Rajilić‐Stojanović et al., 2013). The possible roles of these genera in the pathogenesis of CD may be related with inflammation. However, it should be further evaluated whether their existence causes dysbiosis or consequences of dysbiosis.

*Bacteroides* is one of the most associated genus with CD. To date, especially, in the active period of the disease, alterations in various species belonging to this genus have been reported (Collado et al., 2007, 2009; Sanchez et al., 2010; Schippa et al., 2010). In the studies by Sanchez et al. (2010, 2012) *Bacteroides ovatus* had lower levels in the active period of CD in children. In support of these studies, we found that the relative abundance of *Bacteroides ovatus* increased under GFD treatment. *Bacteroides* is one of the most dominant genera in the human intestinal system, and the *Bacteroides ovatus* is a symbiotic member of the human gastrointestinal tract with immunoregulatory effects. Furthermore, *Bacteroides ovatus* was responsible for increased serum antibody responses in inflammatory bowel diseases (Saitoh et al., 2002) or considering a possible diagnostic microorganism; (Wiredu Ocansey et al., 2023) however, it has not been established whether *Bacteroides ovatus* directly affects the pathogenesis of the CD. On one hand, it has been shown that *Bacteroidetes* was more numerous in the group of CD patients on GFD (Kaliciak et al., 2022). On the other hand, *Bacteroides ovatus* was more abundant in nonceliac controls (Sanchez et al., 2010). In a recent study, *Bacteroides ovatus* strains induce a shift in mice from being poor producers of IgA to becoming high producers of IgA (Yang et al., 2020). This could help the mucosal healing in CD. There is evidence showing decreased mucosal inflammation of the *Bacteroides ovatus* ATCC 8384 monotherapy in marine colitis model (Ihekweazu et al., 2019) and promotion of IL‐22 production via dendritic cell differentiation (Ihekweazu et al., 2021).

In this study, the relative abundance of *Lachnospiraceae UCG‐004* decreased under GFD. Given the fact that *Lachnospiraceae* species are among the main producers of short‐chain fatty acids, they are also associated with different gastrointestinal diseases including inflammatory bowel disease and Crohn's disease (Vacca et al., 2020). In a recent study, the *Lachnospiraceae UCG‐004* has been increased in patients with cervical cancer (Wang, Wang, et al., 2019) and decreased in advanced coronary artery disease patients (Toya et al., 2020). Therefore, the role of the taxa on inflammation may be the explanation for decreased *Lachnospiraceae UCG‐004* abundance under GFD. Another explanation can be the altered dietary composition of the CD patients. Altered levels of the *Lachnospiraceae UCG‐004* were shown in response to a 6‐day lifestyle‐based intervention including increased whole foods, plant‐based foods, and decreased sugar, salt, and oil (Ahrens et al., 2021). However, in a twin study, there was no relationship between the nutrients and *Lachnospiraceae UCG‐004* even though the intratwin *Lachnospiraceae UCG‐004* abundances were different (Matsumoto et al., 2021). Supported that we did not find any association between the nutrient intakes and *Lachnospiraceae UCG‐004* in both CD.P and CD.T groups.

Most studies have pointed out that children with CD have reduced *Lactobacillus* and *Bifidobacterium* species compared to the controls (Collado et al., 2008, 2009). The inflammation‐reducing activities of strains belonging to these species are also behind probiotic‐based therapies (Håkansson et al., 2019). In this study, no significant differences were found between the relative abundance of *Lactobacillus* and *Bifidobacterium* genera; however, when the actual abundances were evaluated, higher counts of *Lactobacillus* (CD.P: 35.5 ± 31.12 vs. CD.T: 18.4 ± 3.98, *p* > .05) and *Bifidobacterium* (CD.P: 2417.4 ± 1959.78 vs. CD.T: 1521.5 ± 2590.92, *p* > .05) were observed before GFD. It is noteworthy that dietary alterations after GFD can be a decreasing factor for these species as discussed at following of the discussion.

The gut microbiome has significant functions in human physiology. It synthesizes vitamins, aids in food digestion, maintains the structural integrity of the gut mucosal barrier to prevent the growth of harmful pathogens, facilitates communication between the gut and brain, and enhances the immune system to promote overall health (Sacchetti & Nardelli, 2020). Dietary composition has certain effects on gut microbiota. Microbiota‐accessible carbohydrates (MACs) refer to dietary carbohydrates that escape digestion but can be metabolized by members of the microbiota, as well as all carbohydrate sources that can be produced by the host or host‐derived microbiota. Dietary MACs can be of plant or animal origin, are utilized by the gut microbiota as an energy source and are known to be substrates for the synthesis of short‐chain fatty acids (SCFAs). Acetate, propionate, and butyrate, which are formed by metabolizing carbohydrates, are known to contribute to the maintenance of intestinal health by preventing inflammation and apoptosis as well as being used as energy sources for host cells (Ayakdaş & Ağagündüz, 2023). *Bacteroides*, *Prevotella*, *Parabacteroides*, and *Alistipes* genera belonging to the Bacteroidetes phylum are known to utilize carbohydrates intensively in the human gut microbiota (Kaoutari et al., 2013). Among Firmicutes phylum especially Ruminococcaceae family mostly utilize insoluble fiber sources (wheat bran, etc.) or resistant starch (Gu et al., 2010; Ze et al., 2012).

In several studies, gut microbiota alterations occurred in response to GFD in healthy subjects (De Palma et al., 2009; Hansen et al., 2018). In this study, we found significant associations between several taxa and especially macronutrient consumption in both CD.P and CD.T groups. However, the associations did not maintain as same, suggesting that utilization of the nutrients may be altered with differences in the microbiota composition under GFD. There is limited study to examine associations between nutrients and microbiota alterations under GFD in CD patients. However, in a study, meat and bean consumptions of CD patients were associated with decreased *Firmicutes* and *Lactobacillus* (Soheilian‐Khorzoghi et al., 2022). In the present study, we found a negative relationship between fat intake of the children under GFD and the relative abundances of the *Bifidobacterium* genus (Figure 7b) and *Bifidobacterium adolescentis* (Figure 7d). Supported that high‐fat diets are linked with decreased *Bifidobacterium* in conditions such as diabetes and obesity (Chen et al., 2023).

In the present study, positive correlations between fiber intake and *Ruminococcaceae UCG.005* genera, *Anaerostipes hadrus*, *Parabacteroides distasonis* species, and negative correlations between the relative abundance of *Erysipelatoclostridium ramosum* species were found (Figure 7b,d). Many species of the Ruminococcaceae family are known to degrade carbohydrates (Flint et al., 2012). Likewise, *Anaerostipes hadrus* is a butyrate producer that has been found to increase with the resistant starch content of the diet (Trachsel et al., 2019). *Parabacteroides distasonis* has been reported to be protective against obesity and metabolic dysfunction and to modulate the host's gut microbiota through succinate and secondary bile acid production (Wang, Liao, et al., 2019). Therefore, it was thought that the increase of these genera and species contributing to butyrate production in children after diet should be supported by fiber intake. Since it is known that the relative abundance of the Erysipelotrichaceae family increases in inflammation‐mediated gastrointestinal system diseases (Kaakoush, 2015), the decrease in their relative abundance with fiber intake may be considered positive.

The relative abundance of the genus *Lachnoclostridium* in the microbiota of children following a gluten‐free diet was found to be inversely correlated with carbohydrate intake and inversely correlated with fat, protein, vegetable protein, and iron intake (Figure 7b). This genus belonging to the Lachnospiraceae family has been shown to induce obesity in sterile ob/ob mice and to be involved in the development of diabetes (Kameyama & Itoh, 2014). In another study, it was reported that the relative abundance of Lachnoclostridium contributed to dysbiosis and was positively correlated with IL‐6, TNF‐α and cholesterol levels in mice with a high‐fat diet and nonalcoholic fatty liver disease model (Tang et al., 2018). Therefore, in this study, it was observed that fat intakes, which could not be reduced with the gluten‐free diet compared to before, still continued to increase the relative abundance of a genus that can trigger inflammation. Therefore, it can be concluded that fat and protein intakes should be reduced, and carbohydrate sources should be diversified. In support of this view, it has been shown that increasing the carbohydrate and fiber content of the diet from alternative cereals (such as quinoa, brown rice, gluten‐free rye flour, pea flour, and oats) in adult individuals receiving low‐gluten dietary interventions resulted in reductions in the Lachnospiraceae family (Hansen et al., 2018).

It has been shown that high‐fat diets (40% and above) can cause a decrease in the Bacteroidetes phylum and an increase in the Firmicutes and Proteobacteria phyla in mice (Ramos‐Romero et al., 2018). Germ‐free mice are resistant to the metabolic consequences of dietary fats, so metabolic dysfunctions due to fat intake may occur through the microbiota (Rabot et al., 2010). Increased dietary saturated fatty acid content has been shown to increase fat‐induced inflammation by reducing phylogenetic diversity (Martinez‐Guryn et al., 2018). This study found that *Tyzzerella 4* was associated with fat intake in the same direction in the CD.P group (Figure 7a). In one study, the genus *Tyzzerella 4* was associated with an increased lifetime risk of cardiovascular disease (Kelly et al., 2016) and in another study with oxidative stress (Zhao, Ou, et al., 2018). In CD, gluten is known to cause oxidative stress and trigger inflammation in the intestine, leading to damage (Ferretti et al., 2012). In the present study, it was found that the genus *Fusicatenibacter* increased with both increased total fat intakes and saturated fat intakes (Figure 7a). It has been reported that the genus *Fusicatenibacter* is influenced by dietary fat intake and its relative abundance increased in rats fed a high‐fat diet in an experimental animal study (Huang et al., 2016). Furthermore, the genus *Fusicatenibacter* has been shown to have an increased relative abundance in patients with the autoimmune disease Hashimoto's thyroiditis and is a biomarker for this disease (Zhao, Feng, et al., 2018). It is interesting that high fat intake contributes to the increase in this genus, as well as its possible association with autoimmune diseases. In this study, the genus *Ruminococcus gnavus group* was also found to be associated with dietary fat intakes (Figure 7a). This genus has recently been reported in the literature as a genus associated with inflammatory bowel diseases (Hall et al., 2017; Schirmer et al., 2019). Recently, *Ruminococcus gnavus* species has been shown to produce and secrete a complex glucorhamnan polysaccharide that induces inflammation in Crohn's disease by contributing to TNF‐α release (Henke et al., 2019).

In the study, it was found that dietary protein intake was positively correlated with the genus *Holdemanella* belonging to the family Erysipelotrichaceae, while plant protein intake was inversely correlated with the genus *Erysipelatoclostridium* and *Erysipelatoclostridium ramosum* (Figure 7a). It was also found that an increase in animal protein intake caused a decrease in *Akkermansia* genus and *Akkermansia muciniphila* (Figure 7a,c). The Erysipelotrichaceae family has been reported to have increased relative abundance in inflammation‐mediated gastrointestinal tract diseases (Kaakoush, 2015). At the same time, in a study comparing the groups given milk protein and soy protein by simulating Western‐style nutrition in experimental animals, it was shown that the relative abundance of Erysipelotrichaceae was higher in the group given milk protein (Butteiger et al., 2015). In recent years, it has been reported that *Akkermansia* genus and *Akkermansia muciniphila*, which are closely related to changes in body weight, also play a role in modulating the host immune system (Ansaldo et al., 2019). Thus, in the presence of CD, the relative abundance of Erysipelotrichaceae may be increased in relation to total protein intakes; however, the type of protein is important.

The strength of the present study is the prospective follow‐up of the cohort and controlled dietary treatment. This report represents the 6‐month microbial alterations of the gut within the negative serology. The follow‐up decreased the bias and provided genetic and environmental control. The relatively small sample, and the lack of control group may be the main limitations of this study. However, because genetic and environmental factors highly shape the gut microbiota, it is a great challenge to compare children with CD and healthy pairs. In most studies, comparisons of CD patients under GFD with a control group who did not have CD, however, needed endoscopy for some reason (functional dyspepsia, gastroesophageal reflux, etc.) (Cheng et al., 2013; Sanchez et al., 2013; Schippa et al., 2010). In addition to the methodological differences between the studies, the dietary pattern, which was not evaluated in almost any study, may also be effective in not determining a CD‐specific gut microbiota. In this study, patients were followed longitudinally, dietary patterns were evaluated, and associated microbial changes are thought to ensure that the differences obtained before and after the diet are reliable. In the present study, the 16 s rRNA amplicon sequencing method was chosen for the microbiota analysis. This method provides most of the results among studies in patients with CD (Sacchetti & Nardelli, 2020). It is important to acknowledge several limitations when comparing to the whole genome shotgun sequencing. Whole genome sequencing offers improved identification of bacterial species, heightened detection of genetic variation, and greater gene prediction capabilities (Ranjan et al., 2016). However, the 16 s rRNA is a short and conserved gene specific to bacterial genus and the species, economical, and widely used option. The V4/V3/V3‐V4/V4‐V5 partial regions have been shown as good choices for bacterial analysis (Kim et al., 2011; Yang et al., 2016).

## 5 CONCLUSIONS

The present study qualified the gut microbiota of children with CD under a well‐controlled dietary follow‐up. In conclusion, 6 months under GFD treatment provided negative serology. However, it was insufficient to observe distinct microbiota pattern in CD patients, although resulted in a reliable difference in gut microbiota. Presence or absence situation of minor bacteria may have a role in CD pathogenesis. We showed different associations between gut microbiota and diet. These may be a reason for the gut microbiota alterations as well. Future studies may be focused on the following directions: (1) The minor bacteria alterations should be investigated whether a cause or consequence in CD. (2) Possible diagnostic, prognostic role of the *Bacteroides ovatus* can be investigated since the evidence on its anti‐inflammatory effects growing. (3) The genus *Lachnospiraceae* should be studied in patients with CD to enlighten the mechanistic role of its species on CD. (4) Further larger longitudinal studies are needed to provide data on how environmental factors such as dietary intake can interact with gut microbiota and inflammation in CD.

## AUTHOR CONTRIBUTIONS

**Yasemin Ertaş Öztürk:** Conceptualization (equal); data curation (lead); formal analysis (lead); investigation (lead); methodology (equal); writing – original draft (lead); writing – review and editing (equal). **Efsun Karabudak:** Conceptualization (equal); methodology (equal); project administration (lead); writing – review and editing (equal). **Ödül Eğritaş Gürkan:** Conceptualization (equal); data curation (supporting); methodology (equal); project administration (supporting); writing – review and editing (supporting). **Buket Dalgıç:** Data curation (supporting); project administration (supporting); writing – review and editing (supporting).

## FUNDING INFORMATION

This study was supported by Gazi University, Scientific Research Projects Unit with project number 47/2019‐02.

## CONFLICT OF INTEREST STATEMENT

The authors declare no conflict of interest.

## ETHICAL APPROVAL

The study protocol was approved by the Clinical Research Ethics Committee of Gazi University (date: 22/01/2018 and number: 24074710‐8/42).

## CONSENT STATEMENT

Written consents of all parents were taken and ethical approval was obtained from Gazi University, Clinical Research Ethics Committee.

## Data Availability

The datasets generated during and/or analyzed during the current study are available from the corresponding author on reasonable request.
